# Robotic-assisted total knee arthroplasty is not associated with increased risk of postoperative deep vein thrombosis

**DOI:** 10.1186/s40634-023-00628-6

**Published:** 2023-06-29

**Authors:** Junya Itou, Umito Kuwashima, Masafumi Itoh, Ken Okazaki

**Affiliations:** Department of Orthopaedic Surgery, Tokyo Women’s Medical University, 8-1 Kawada-Cho, Shinjuku-Ku, Tokyo, 162-8666 Japan

**Keywords:** Total knee arthroplasty, Deep vein thrombosis, Robotics

## Abstract

**Purpose:**

Prolongation of operation time due to registration and pin insertion has been reported with robotic-assisted total knee arthroplasty (RATKA), and there has been concern about an increase in the postoperative incidence of deep vein thrombosis (DVT). In this study, we compared the incidence of DVT after RATKA with that after conventional manual TKA (mTKA).

**Methods:**

This consecutive retrospective series included 141 knees that underwent primary TKA using the Journey II system. The CORI robot was used. There were 60 RATKAs and 81 mTKAs. Doppler ultrasound was performed in all patients on postoperative day 7 to determine whether DVT was present.

**Results:**

The operation time was longer in the RATKA cohort (99.5 min vs 78.0 min, *p* < 0.001). The overall incidence of DTV was 43.9% (62/141 knees), all of which were asymptomatic. There was no significant difference in incidence of DVT between RATKA and mTKA (50.0% vs 39.5%, *p* = 0.23). Use of the robot did not affect the incidence of DVT following TKA (odds ratio 1.02, 95% confidence interval 0.40–2.60; *p* = 0.96).

**Conclusion:**

The incidence of DVT was not significantly different between RA-TKA and mTKA. Multiple logistic regression indicated that RATKA is not associated with increased risk of postoperative DVT.

**Level of evidence:**

IV.

## Introduction

Robotic-assisted total knee arthroplasty (RATKA) has excellent advantages, including accurate placement of the prosthesis, precise bony cuts, less soft tissue invasion, real-time intraoperative assessment of ligament balance, and better short- to long-term postoperative functional outcomes and patient satisfaction [1, 5, 12, 15, 25]. However, recent systematic reviews comparing RATKA with conventional manual TKA (mTKA) have identified complications and downsides [25]. Well-known complications include pin-hole fracture, pin-related infection, iatrogenic soft tissue and bony injury, and excessive blood loss with the downside of a longer operation time [11, 16].

Deep vein thrombosis (DVT) is a widely recognized major complication in patients undergoing TKA [2, 8, 24]. Many studies have investigated factors associated with the incidence of DVT, the most well-known of which are older age, female sex, higher body mass index (BMI), bilateral surgery, and cemented fixation [23]. Postoperative DVT is assumed to be a negative event caused by increased surgical invasiveness and a longer operation time [26], but there is still limited information on DVT after RATKA. Prolongation of operation time as a result of registration and pin insertion has been reported with RATKA [11], and there is concern about an increased incidence of DVT [13]. Given that factors contributing to the incidence of DVT, such as soft tissue invasion and operation time, are different between RATKA and mTKA, it would be rational to evaluate the incidence of DVT after RATKA. Accurate assessment of the incidence of DVT allows surgeons to accurately inform patients of the risks and benefits when counseling them about the procedure.

The purpose of this study was to compare the incidence of DVT after RATKA with that after mTKA. Our hypothesis was that the incidence of DVT after RATKA would be affected by the prolonged operation time.

## Methods

### Study design and participants

This consecutive retrospective series included 141 knees that underwent primary TKA using the Journey II system (Smith & Nephew, Memphis, TN, USA) [9, 10, 17] at our institution between January 2020 and December 2022. Since December 2021, we have used the CORI robot (Smith and Nephew, London, UK) for TKA [6]. All TKAs were performed in patients with a diagnosis of osteoarthritis, osteonecrosis, or rheumatoid arthritis. No knees with posttraumatic arthritis were present in this retrospective series. There were 60 RATKAs and 81 mTKAs.

This study was approved by our hospital ethics committee (approval number 4952). Informed consent was obtained via the opt-out route in view of the retrospective observational nature of the research.

### Surgical technique and postoperative rehabilitation

All of the surgeries were performed using a tourniquet by any of four experienced knee surgeons who were all knowledgeable about the implant and the implantation techniques used. The tourniquet inflation pressure was based on systolic blood pressure [20]. The tourniquet was inflated before the skin incision and loosened after application of a bandage to the limb. The decision regarding whether to perform bi-cruciate stabilized or cruciate-retaining TKA was at the discretion of the surgeon. All TKAs were cemented and resurfaced with a cemented dome-shaped polyethylene patella.

All surgeries were performed under general anesthesia, and additional regional anesthesia was added under ultrasound guidance at the discretion of the anesthesiologist. A drainage tube was not placed in all cases. Tranexamic acid 1000 mg was administered intravenously before the tourniquet was released. Antiplatelet agents or anticoagulants that had been administered preoperatively were withdrawn only on the day of surgery, and oral intake was resumed on the first postoperative day.

All patients received standard mechanical prophylaxis against DVT, namely, use of elastic stockings and an intermittent pneumatic compression device in the early postoperative period [8, 21]. Patients were permitted to ambulate with full weight-bearing as pain permitted from the first postoperative day and underwent rehabilitation without restriction of range of motion.

### Assessment for venous thromboembolism

Venous thromboembolism (VTE) includes both DVT and pulmonary embolism (PE) [23, 24]. D-dimer plasma levels were assessed before surgery to rule out preoperative DVT. Patients with a preoperative D-dimer cut-off value of 1.0 μg/mL [14] who underwent whole-leg Doppler ultrasound and were noted to have DVT were not diagnosed to have new DVT if the DVT was found at the same site postoperatively.

Doppler ultrasound was performed on day 7 after surgery in all patients to determine whether postoperative DVT was present. DVT was assessed by whole-leg Doppler ultrasound, and the location of any thrombus was recorded. Compression and color Doppler imaging in B-mode ultrasonography were performed. Proximal DVT was defined as DVT occurring in the popliteal vein or higher. Whether the thrombus was attached to the vein wall or free-floating was also investigated. All Doppler ultrasound procedures were performed by the same team of experienced ultrasonographers. The ProSound F75 (Hitachi Aloka Medical, Tokyo, Japan) was used for all cases.

If PE was suspected based on clinical signs [8, 23], such as chest pain, breathlessness, and desaturation, contrast-enhanced computed tomography was performed.

### Complications and clinical assessment

Complications occurring up to 90 days following TKA were retrospectively analyzed using the patients’ medical records. Postoperative mobilization time was defined as the first day on which the patient ambulated or performed standing foot exercises after surgery.

Risk stratification was performed using age and the American Society of Anesthesiologists (ASA) score, which refers to the classification of the patient’s physical status before surgery. Patients classified into the following three risk categories: high risk (age ≥ 75 years and ASA > 2), intermediate risk (age ≥ 75 years or ASA > 2), and low risk (age < 75 years and ASA ≤ 2) [3].

### Statistical analysis

Descriptive statistics are reported as the median (range), the count (percentage), or the mean and standard deviation. The distribution of continuous variables was assessed for normality by visual inspection (histograms) and the Shapiro–Wilk test. The chi-squared test was used to determine the statistical significance of between-group differences in categorical variables and the Wilcoxon signed-rank test to evaluate the significance of between-group differences in continuous variables. Multiple logistic regression was used to assess the association of demographic factors with the risk of DVT. All statistical analyses were performed using JMP software version 16 (SAS Institute Inc., Cary, NC). A *p*-value < 0.05 was considered statistically significant.

A post hoc power analysis was conducted using G*Power (version 3.1.9.6). When the level of significance was set at 0.05, the sample sizes of the study groups (*n* = 60 and *n* = 81) had a power of 89%.

## Results

The patient demographics are shown in Table 1. The operation time was longer in the RATKA group than in the mTKA group (99.5 min vs 78.0 min, *p* < 0.001). There were no serious complications such as pin-related infection, pin-hole fracture, or fatal PE during the observation period.

**Table 1:** Patient demographics and clinical characteristics

Group	RATKA (*n* = 60)	mTKA (*n* = 81)	*p*-value	SMD
Age at surgery, years, median [range]	76.0 [68.0–80.0]	73.0 [64.0–78.0]	**0.04**	0.36
Male sex, n (%)	16 (26.7)	21 (25.9)	1.00	0.01
Body mass index^a^, median [range]	26.1 [24.1–28.3]	25.9 [24.6–29.1]	0.61	0.16
Operated side: right (%)	25 (41.7)	47 (58.0)	0.06	0.33
Preoperative treatment, n (%)	A: 47 (78.3)	A: 58 (71.6)	0.16	0.33
	B: 4 (6.7)	B: 14 (17.3)		
	C: 9 (15.0)	C: 9 (11.1)		
Risk category, n (%)	Low: 29 (48.3)	Low: 36 (44.4)	0.36	0.25
	Intermediate: 22 (36.7)	Intermediate: 38 (46.9)		
	High: 9 (15.0)	High: 7 (8.6)		
Operation time, min, median [range]	99.5 [92.0–111.0]	78.0 [73.0–84.0]	** < 0.001**	1.74
Time to postoperative mobilization, days, median [range]	1.0 [1.0–2.0]	1.0 [1.0–2.0]	0.19	0.15
DVT, n (%)	30 (50.0)	32 (39.5)	0.23	0.21

^a^Calculated as kg/m^2^. A, none; B, antiplatelet agents; C, anticoagulants; DVT, deep vein thrombosis; mTKA, RATKA, SMD, standardized mean difference. Bold values denote statistical significance at *p* < 0.05

### Incidence and characteristics of VTE

The overall incidence of VTE was 43.9% for DVT (62/141 knees) and 0% for PE (0/141 knees). There was no significant difference in the incidence of DVT between RA-TKA and mTKA (50.0% vs 39.5%,* p* = 0.23, Table 1).

Table 2 show the details of the postoperative DVTs that occurred, all of which were asymptomatic. Notably, most of the DVTs (82.2%) occurred in the soleal vein of the affected limb but 19.5% occurred in the soleal vein on the unaffected limb.

**Table 2:** Details of the deep vein thromboses that occurred postoperatively

Affected side	SoV	PeV	PTV	PV	FV	GV
Incidence of DVT (free-floating), %	82.2	33.0	19.3	9.6 (4.8)	4.8 (1.6)	6.4
Unaffected side	SoV	PeV	PTV	PV	FV	GV
Incidence of DVT (free-floating), %	19.3	3.2	1.6	1.6	1.6	1.6

*DVT* Deep vein thrombosis, *FV* Femoral vein, *GV* Gastrocnemius vein, *PeV* Peroneal vein, *PTV* Posterior tibial vein, *PV* Popliteal vein, *SoV* Soleal vein

Floating thrombi were found in four knees (three in the popliteal vein and one in the femoral vein) (Table 2).

### Risk factors for postoperative DVT

Potential risk factors for postoperative DVT, namely, age at surgery, sex, BMI, preoperative antithrombotic therapy, operation time, time to postoperative mobilization, risk category, and robotic assistance were examined in a multiple logistic regression model with adjustment for confounders. The multiple logistic regression revealed that none of these factors were significantly associated with the incidence of DVT following TKA (Table 3). In particular, use of the robot did not affect the incidence of DVT (odds ratio 1.02, 95% confidence interval 0.40–2.60; *p* = 0.96).

**Table 3:** Results of multiple logistic regression to identify risk factors for postoperative deep vein thrombosis

Factor	Odds ratio	95% CI lower	95% CI upper	*p*-value
Age at surgery	1.04	0.99	1.09	0.06
Female sex	0.75	0.32	1.74	0.50
Body mass index	0.96	0.87	1.05	0.38
Preoperative treatment [C]	0.55	0.18	1.69	0.29
Preoperative treatment [B]	1.29	0.42	3.90	0.65
Operation time	1.02	0.99	1.05	0.21
Time to postoperative mobilization	1.01	0.74	1.38	0.94
Robotic assistance	1.02	0.40	2.60	0.96
Risk category	0.85	0.39	1.80	0.66

*B* antiplatelet agents, *C* anticoagulants, *CI* Confidence interval, *DVT* Deep vein thrombosis

## Discussion

The most important finding in this study was that the incidence of DVT after RATKA was not significantly different from that after mTKA despite RATKA having a longer operation time. As in previous studies [11, 15], there was a difference in operation time of about 20 min, but the effect of the longer operation time required for RATKA on the risk of DVT was considered small.

There are several potential interpretations of the results of this study. Our hypothesis that the incidence of DVT would be higher after RATKA because of its longer operation time was not supported by the results. The advantages of RATKA may have compensated for the longer operation time and minimized the occurrence of postoperative DVT. RATKA is less invasive to soft tissues [5], which may be advantageous for early discharge and resumption of activities of daily living [16]. Furthermore, RATKA does not require insertion of an intramedullary guiding rod for preparation of femoral bone, which may reduce the invasiveness in terms of the venous circulation. There has been a study suggesting that the incidence of DVT is lower with RATKA than with mTKA [16] but a shortcoming of that research was that it used International Classification of Disease codes and did not directly assess DVT in detail. The strengths of our study were that it was a comparison of RATKA and mTKA at the same institution and all patients underwent postoperative whole-leg Doppler ultrasound to accurately assess DVT, including asymptomatic cases. In addition, to our knowledge, this was the first study to evaluate the distribution of DVT after RATKA in the lower extremities on both the healthy and operated sides.

Our multiple logistic regression did not reveal any factors that showed a significant association with the incidence of DVT. In a previous systematic review, older age, female sex, higher BMI, and longer operation time were considered to be risk factors for VTE [26]. However, these risk factors are known to be controversial [24, 26] and it is unclear whether they have a direct or indirect influence on the incidence of symptomatic and asymptomatic DVT and PE. All the DVTs detected in our present study were asymptomatic, which may not have been the case in the previous studies.

Chemoprophylaxis with low-molecular-weight heparin or direct oral anticoagulants (DOACs) has been widely used to protect against VTE [21]. In particular, the newly developed DOACs, which are direct Factor Xa inhibitors and include rivaroxaban, apixaban, and edoxaban, are frequently prescribed in the cardiovascular field and were used in just over 10% of the cases in this study. VTE prophylaxis after TKA with DOACs has been reported [4, 18]. However, high drug costs and complications such as bleeding were concerns. Furthermore, in the present study, the frequency of postoperative VTE following TKA was not significantly lower in the group that received DOACs.

This study has several limitations. First, a retrospective design could be a source of bias. The patient background characteristics were comparable between the study groups, though the RATKA group was significantly older. Older age is a risk factor for DVT and may explain the higher incidence of DVT in the RATKA group (50.0% vs. 39.5%, *p* = 0.23). Second, the learning curve required for RATKA may have affected our results in terms of operation time. There have been reports of continued improvement in operation times when using RATKA [1, 11], and further shortening of the time taken to perform RATKA could further reduce the incidence of DVT. Third, the routine use of a tourniquet can increase the risk of DVT [7]. However, given the conflicting results in the literature regarding DVT [19], the use of tourniquets during TKA is controversial. Finally, DVT that developed after postoperative day 7 might not have been recognized. Most asymptomatic DVTs after TKA are estimated to occur within the first 4 postoperative days [22]. Therefore, postoperative day 7 was considered to be the appropriate time to perform Doppler ultrasound [7]. However, asymptomatic DVTs after TKA do not all occur within the first 7 postoperative days [18], so further prospective studies that reveal the natural history of asymptomatic DVT after RATKA after postoperative day 7 are needed.

## Conclusion

The incidence of DVT was not significantly different between RA-TKA and mTKA. Multiple logistic regression indicated that RATKA is not associated with increased risk of postoperative DVT.

## Data Availability

The data that support the findings of this study are available from the corresponding author upon reasonable request.

## Abbreviations

BMI: Body mass index
DVT: Deep vein thrombosis
mTKA: Manual total knee arthroplasty
PE: Pulmonary embolism
RATKA: Robotic-assisted total knee arthroplasty
VTE: Venous thromboembolism

## References

[1] Bolam SM, Tay ML, Zaidi F, Sidaginamale RP, Hanlon M, Munro JT, Monk AP (2022). Introduction of ROSA robotic-arm system for total knee arthroplasty is associated with a minimal learning curve for operative time. J Exp Orthop.

[2] Chang MJ, Song MK, Kyung MG, Shin JH, Chang CB, Kang SB (2018). Incidence of deep vein thrombosis before and after total knee arthroplasty without pharmacologic prophylaxis: a 128-row multidetector CT indirect venography study. BMC Musculoskelet Disord.

[3] Dale H, Børsheim S, Kristensen TB, Fenstad AM, Gjertsen JE, Hallan G, Lie SA, Furnes O (2020). Perioperative, short-, and long-term mortality related to fixation in primary total hip arthroplasty: a study on 79,557 patients in the -Norwegian Arthroplasty Register. Acta Orthop.

[4] Fuji T, Fujita S, Tachibana S, Kawai Y (2010). A dose-ranging study evaluating the oral factor Xa inhibitor edoxaban for the prevention of venous thromboembolism in patients undergoing total knee arthroplasty. J Thromb Haemost.

[5] Hampp EL, Sodhi N, Scholl L, Deren ME, Yenna Z, Westrich G, Mont MA (2019). Less iatrogenic soft-tissue damage utilizing robotic-assisted total knee arthroplasty when compared with a manual approach: a blinded assessment. Bone Joint Res.

[6] Honecke T, Schwarze M, Wangenheim M, Savov P, Windhagen H, Ettinger M (2022). Noise exposure during robot-assisted total knee arthroplasty. Arch Orthop Trauma Surg.

[7] Huang CR, Pan S, Li Z, Ruan RX, Jin WY, Zhang XC, Pang Y, Guo KJ, Zheng X (2021). Tourniquet use in primary total knee arthroplasty is associated with a hypercoagulable status: a prospective thromboelastography trial. Int Orthop.

[8] Itou J, Kuwashima U, Itoh M, Okazaki K (2021). No difference in the incidence or location of deep venous thrombosis according to use of pharmacological prophylaxis following total knee arthroplasty. BMC Musculoskelet Disord.

[9] Kuwashima U, Hamai S, Okazaki K, Ikebe S, Higaki H, Mizu-Uchi H, Akasaki Y, Murakami K, Iwamoto Y (2016). Contact stress analysis of the anterior tibial post in bi-cruciate stabilized and mobile-bearing posterior stabilized total knee arthroplasty designs. J Mech Behav Biomed Mater.

[10] Kuwashima U, Mizu-Uchi H, Okazaki K, Hamai S, Akasaki Y, Murakami K, Nakashima Y (2017). Three-dimensional analysis of accuracy of patient-matched instrumentation in total knee arthroplasty: evaluation of intraoperative techniques and postoperative alignment. J Orthop Sci.

[11] Marchand KB, Ehiorobo J, Mathew KK, Marchand RC, Mont MA (2022). Learning curve of robotic-assisted total knee arthroplasty for a high-volume surgeon. J Knee Surg.

[12] Mullaji AB, Khalifa AA (2022). Is it prime time for robotic-assisted TKAs? A systematic review of current studies. J Orthop.

[13] Mundi R, Nucci N, Wolfstadt J, Pincus D, Chaudhry H (2023). Risk of complications with prolonged operative time in morbidly obese patients undergoing elective total knee arthroplasty. Arthroplasty.

[14] Niimi R, Hasegawa M, Shi DQ, Sudo A (2012). The influence of fondaparinux on the diagnosis of postoperative deep vein thrombosis by soluble fibrin and D-dimer. Thromb Res.

[15] Nogalo C, Meena A, Abermann E, Fink C (2022). Complications and downsides of the robotic total knee arthroplasty: a systematic review. Knee Surg Sports Traumatol Arthrosc.

[16] Ofa SA, Ross BJ, Flick TR, Patel AH, Sherman WF (2020). Robotic total knee arthroplasty vs conventional total knee arthroplasty: a nationwide database study. Arthroplast Today.

[17] Okazaki K (2022). Adopting the joint line theory for bone resection in cruciate-retaining total knee arthroplasty to prevent flexion gap tightness. Orthop Surg.

[18] Sasaki H, Ishida K, Shibanuma N, Tei K, Tateishi H, Toda A, Yamashiro Y, Matsumoto T, Kuroda R, Kurosaka M (2014). Retrospective comparison of three thromboprophylaxis agents, edoxaban, fondaparinux, and enoxaparin, for preventing venous thromboembolism in total knee arthroplasty. Int Orthop.

[19] Schnettler T, Papillon N, Rees H (2017). Use of a tourniquet in total knee arthroplasty causes a paradoxical increase in total blood loss. J Bone Joint Surg Am.

[20] Sun C, Yang X, Zhang X, Ma Q, Yu P, Cai X, Zhou Y (2022). Personalized tourniquet pressure may be a better choice than uniform tourniquet pressure during total knee arthroplasty: a PRISMA-compliant systematic review and meta-analysis of randomized-controlled trials. Medicine (Baltimore).

[21] Torrejon Torres R, Saunders R, Ho KM (2019). A comparative cost-effectiveness analysis of mechanical and pharmacological VTE prophylaxis after lower limb arthroplasty in Australia. J Orthop Surg Res.

[22] Yamaguchi T, Hasegawa M, Niimi R, Sudo A (2010). Incidence and time course of asymptomatic deep vein thrombosis with fondaparinux in patients undergoing total joint arthroplasty. Thromb Res.

[23] Zeng Y, Si H, Wu Y, Yang J, Zhou Z, Kang P, Pei F, Shen B (2019). The incidence of symptomatic in-hospital VTEs in Asian patients undergoing joint arthroplasty was low: a prospective, multicenter, 17,660-patient-enrolled cohort study. Knee Surg Sports Traumatol Arthrosc.

[24] Zhang J, Chen Z, Zheng J, Breusch SJ, Tian J (2015). Risk factors for venous thromboembolism after total hip and total knee arthroplasty: a meta-analysis. Arch Orthop Trauma Surg.

[25] Zhang J, Ndou WS, Ng N, Gaston P, Simpson PM, Macpherson GJ, Patton JT, Clement ND (2022). Robotic-arm assisted total knee arthroplasty is associated with improved accuracy and patient reported outcomes: a systematic review and meta-analysis. Knee Surg Sports Traumatol Arthrosc.

[26] Zhang ZH, Shen B, Yang J, Zhou ZK, Kang PD, Pei FX (2015). Risk factors for venous thromboembolism of total hip arthroplasty and total knee arthroplasty: a systematic review of evidences in ten years. BMC Musculoskelet Disord.

